# Foliar Water Uptake Supports Water Potential Recovery but Does Not Affect Xylem Sap Composition in Two Salt‐Secreting Mangroves

**DOI:** 10.1111/pce.15332

**Published:** 2024-12-16

**Authors:** Adriano Losso, Alice Gauthey, Stefan Mayr, Brendan Choat

**Affiliations:** ^1^ Hawkesbury Institute for the Environment Western Sydney University Penrith New South Wales Australia; ^2^ Department of Botany Universität Innsbruck/University of Innsbruck Innsbruck Austria; ^3^ Birmingham Institute of Forest Research University of Birmingham Edgbaston UK

**Keywords:** daily course, foliar water uptake, ions, mangroves, tides, xylem sap composition

## Abstract

Mangroves are highly salt‐tolerant species, which live in saline intertidal environments, but rely on alternative, less saline water to maintain hydraulic integrity and plant productivity. Foliar water uptake (FWU) is thought to assist in hydration of mangroves, particularly during periods of acute water deficit. We investigated the dynamics of FWU in *Avicennia marina* and *Aegiceras corniculatum* by submerging and spraying excised branches and measuring leaf water potential (Ψ) at different time intervals. Daily changes in xylem sap composition (ionic concentrations, pH and surface tension) were monitored during 2 days characterised by the presence of morning dew and difference in tides. In both species, FWU occurred over relatively short times, with leaf Ψ recovering from −4.5 MPa to about −1.5 MPa in 120–150 min. At predawn, Ψ was higher (−1.5 MPa) than sea water Ψ, indicating that leaves had been partially rehydrated by absorbed dew. Tides did not affect Ψ, but high tides increased the overall ionic content of xylem sap. The results indicated mangroves are extremely efficient in absorbing non‐saline water via the leaves and restoring the water balance to Ψ higher than seawater. Changes in xylem sap composition, which were strongly influenced by tides, were not affected by observed FWU.

## Summary statement

- Mangroves exhibited rapid foliar water uptake and water potential recovery.
- High tides affected the overall ionic composition of xylem sap, but did not affect early morning dew uptake.

## 1 Introduction

In recent years, absorption of atmospheric water (e.g., dew, rain and snow) via leaves have been reported across a wide range of tree species and ecosystems (e.g., Mayr et al. 2014; Mayr et al. 2019; Steppe et al. 2018; Binks et al. 2019; Schreel, von der Crone, et al. 2019; Schreel, Van de Wal, et al. 2019; Schreel et al. 2020; Hayes et al. 2020; Waseem et al. 2021; Losso et al. 2021; Schaepdryver et al. 2022) and it has been recognised to play a significant role in the alleviation of drought‐induced stresses. In particular, high air humidity during leaf wetting events, such as fog, dew, or drizzle, is of crucial importance when the root zone remains substantially dry (Nadezhdina et al. 2010; Eller, Lima, and Oliveira 2013, 2016; Berry et al. 2019). According to a recent review on foliar water uptake (FWU), up to 85% of plant species can absorb water through their leaves (Berry et al. 2019). The main hypothesis proposed for this process is that, during leaf wetting events, the water potential (Ψ) at the leaf‐atmosphere interface becomes higher than the plant and soil Ψ, thus driving water flows opposite to the direction of plant water transport during transpiration. When these conditions are achieved, water can enter the plant hydraulic network through the cuticle (Eller, Lima, and Oliveira 2013) or thanks to other leaf anatomical features, such as trichomes (Schreel et al. 2020; Waseem et al. 2021), stomata (Eichert and Goldbach 2008; Burkhardt et al. 2012), salt glands (Tan et al. 2013), or cork warts (Bryant et al. 2021).

Amongst all the species known for absorbing water through their leaves, mangroves gained more interest in the past few years (e.g., Steppe et al. 2018; Schreel, Van de Wal, et al. 2019; Hayes et al. 2020; Coopman et al. 2021). Mangroves are fascinating and widespread organisms growing at intertidal zones in tropical and subtropical regions, which are affected by high salinity, high temperatures and local droughts as well as tides and strong winds leading to regular flooding and anoxic soils (Kathiresan and Bingham 2001). In particular, daily tides regularly flood mangroves and expose substrates to high salinity, which creates very strong osmotic pressure on mangroves' hydraulic systems and can thus lead to salt‐induced physiological drought (e.g., Ewers et al. 2004). Under these conditions, FWU can be crucial to maintain hydration in mangroves, as their saline environment induces a permanent state of physiological drought, which affects soil water uptake, transport and use (e.g., Ewers et al. 2004; López‐Portillo, Ewers, and Angeles 2005; Lovelock et al. 2006; Nguyen et al. 2015; Reef et al. 2015). At a [NaCl] of 3% (483 mM Na^+^ and 558 mM Cl^−^), sea water has an osmotic potential of approximately −2.5 MPa (Harvey 1955). Mangroves are thus forced to reduce their Ψ to values lower than those of sea water to avoid water losses to the substrate and allow soil water uptake (Scholander et al. 1965). Hence, access to atmospheric water is crucial for mangroves, as it helps to compensate for limited water uptake at the roots even when soil Ψ is substantially low. In a study on three co‐occurring mangroves, Hayes et al. (2020) suggested FWU to be a common mechanism providing a supplemental water balance strategy. In the mangrove *Avicennia marina* (Forssk.) Vierh., FWU has been recognised to be crucial for maintaining cell turgor (Steppe et al. 2018), enabling recovery of losses in leaf hydraulic conductance upon dehydration (Fuenzalida et al. 2019) and to support turgor‐driven growth spurts in mature trees and saplings (Steppe et al. 2018; Schreel, Van de Wal, et al. 2019).

**Figure 1:**
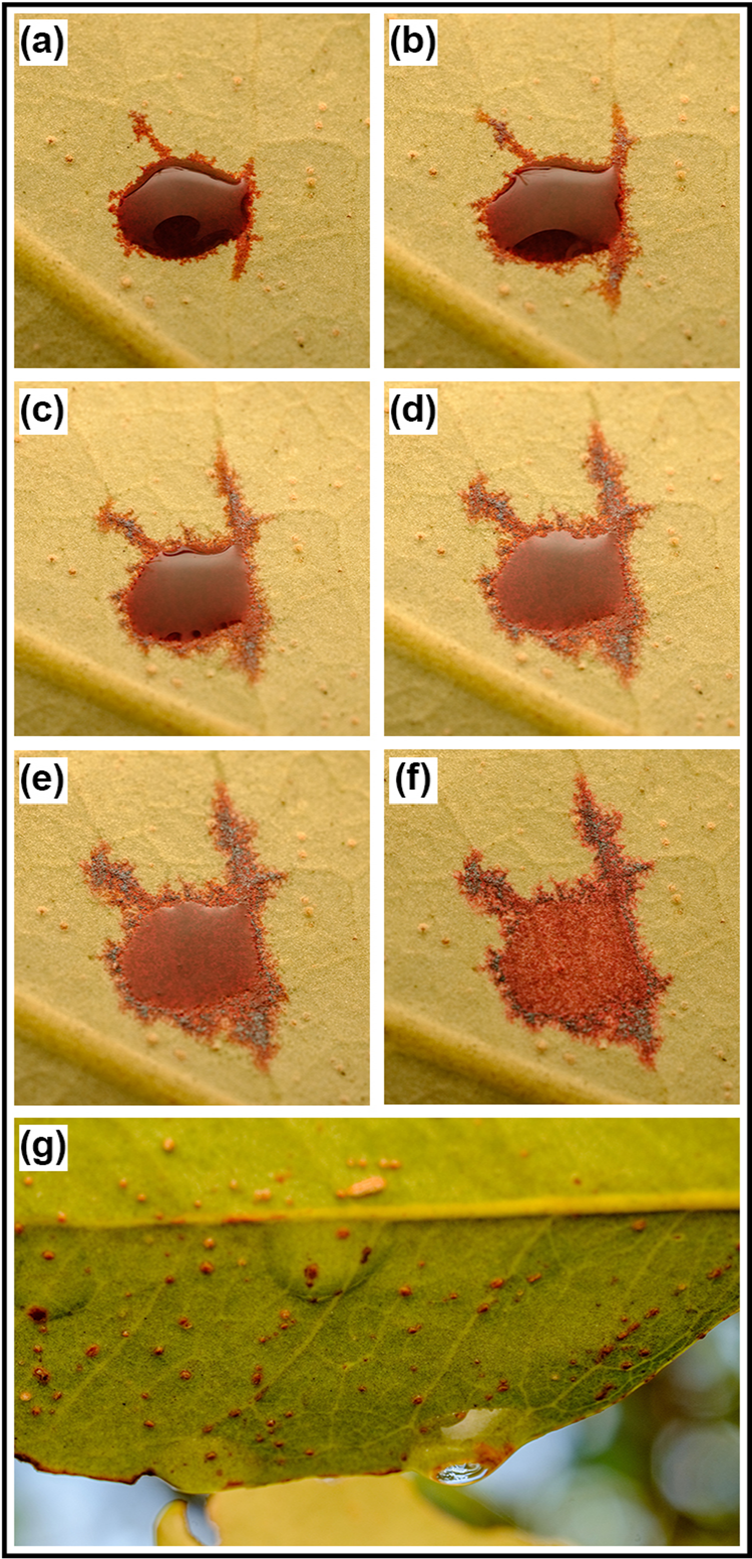
A drop of Safranin being absorbed by the lower surface of a leaf of *Avicennia marina* ssp. *australasica* after 1 (a), 10 (b), 20 (c), 30 (d), 40 (e) and 60 (f) minutes. Naturally occurring dew on the lower surface of leaves of a *A. marina* (g).

**Figure 2:**
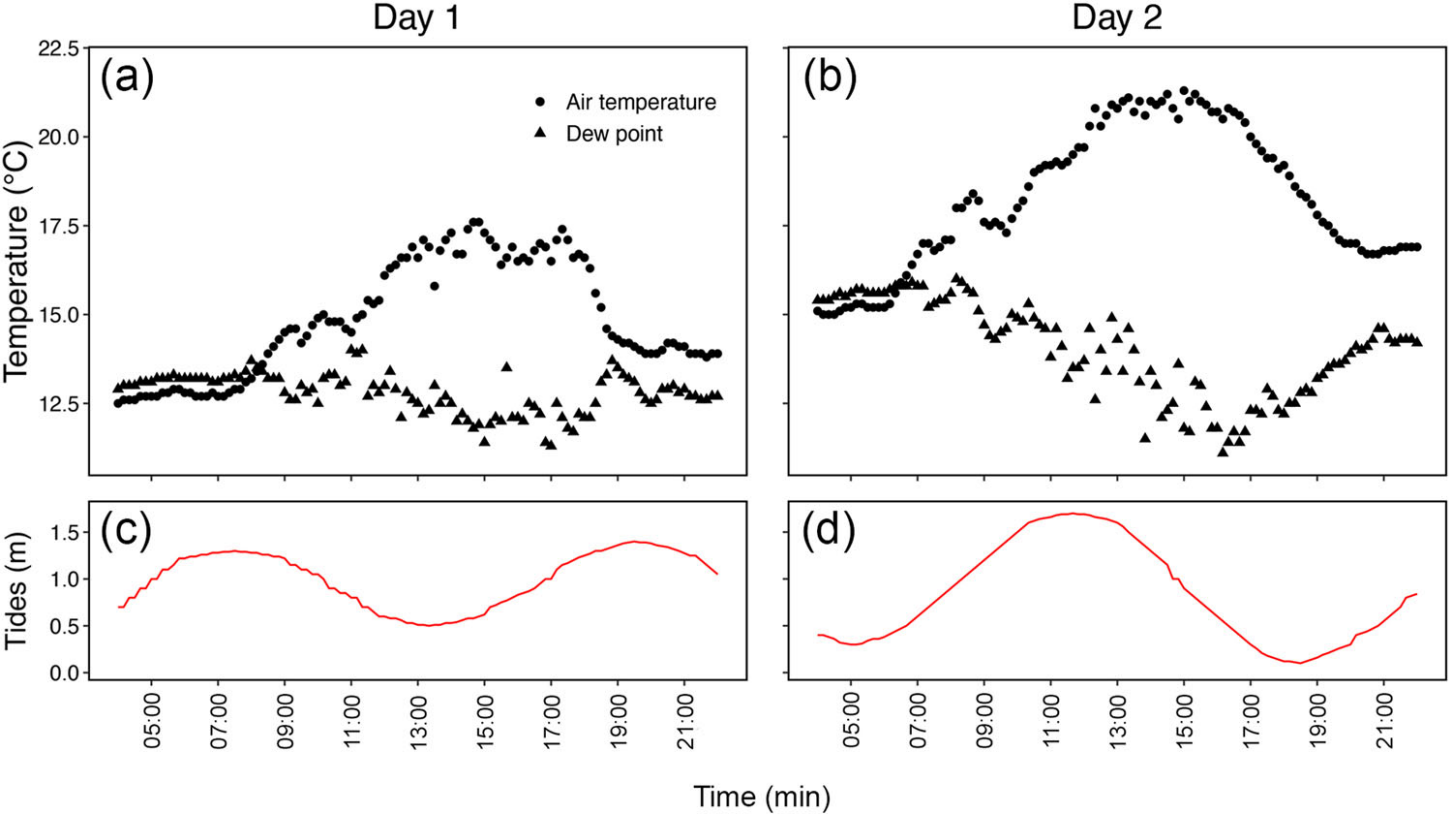
Air temperature (°C; solid circles) and dew point temperature (°C; solid triangles) measured every 10 min from 04:00 to 23:00 hr of Days 1 (27.10.2020; a) and 2 (18.11.2020; b). Tide events (m; red lines) of Days 1 (c) and 2 (d). Temperature data were collected by a weather station near the study site (Terrey Hills AWS Station number 066059; Bureau of Meteorology). [Color figure can be viewed at wileyonlinelibrary.com]

Mangroves are specialised organisms that evolved to grow under highly saline conditions. Their xylem sap is thus characterised by salt concentrations ranging between 0.5 and 380 mM (Ball 1988; Paliyavuth, Clough, and Patanaponpaiboon 2004; López‐Portillo, Ewers, and Angeles 2005), which are much higher than in other land plants (< 3–5 mM). Although all mangrove species filter a certain amount of salt at the roots, specialised physiological traits based on different salt management strategies divide mangroves into two main groups: non‐secretors, which ultrafiltrate sea water directly at the roots, thus preventing high salt concentrations to enter the xylem and secretors, which can withstand higher xylem sap salt concentrations and secrete the excess of salt through specialised glands found at the leaf surface (e.g., Scholander 1968). In a recent study on *A. marina*, the salt‐secreting mechanism has been demonstrated to induce leaf wetting via the deliquescence of accumulated salt at the leaves and support the plant water balance under dry conditions (Coopman et al. 2021). Here, we present a detailed study on the dynamics and the role of FWU in *Avicennia marina* ssp. *australasica* (Walp.) J. Everett and the co‐occurring *Aegiceras corniculatum* (L.) Blanco. *A. marina* is one of the most widespread species of mangroves and arguably one of the most studied mangroves. It is also one of the most tolerant mangroves to salinity, aridity, water temperature and frost frequency (e.g., Morrisey et al. 2010; Jiang et al. 2021; Jiang et al. 2022; Gauthey et al. 2022). *A. corniculatum* is also widespread and is classified as mid‐tolerant to salinity (see Reef and Lovelock 2015). To better understand the implications of FWU, we investigated how quickly leaf Ψ can recover during artificial leaf wetting in both species and if this water is also used for the recovery of stem Ψ. We also selected 2 days with optimal conditions for dew formation on leaves, but opposite daily tide dynamics (Figures 1g and 2). On both days, we extracted xylem sap from branches collected at four different time points (i.e., predawn, midday, afternoon and night), and analysed several xylem sap parameters involved in the regulation of xylem hydraulic efficiency (Na^+^ and K^+^; López‐Portillo, Ewers, and Angeles 2005) and safety (surface tension, γ; Losso et al. 2017), as well as used as a proxy for drought and salinity stress (pH and Ca^2+^; Kader and Lindberg 2010; Losso et al. 2018; Pagliarani et al. 2019). Previous studies (Scholander et al. 1962; Drennan and Pammenter 1982; Ball 1988; Sobrado 2002; López‐Portillo, Ewers, and Angeles 2005) have investigated changes in xylem sap salinity but never considered the influence of neither FWU of predawn dew formation nor tide events. Hence, the main hypotheses of this study were (i) that both mangrove species can recover their leaf and stem Ψ via FWU when exposed to sufficient external water sources, and (ii) that this water can affect the composition of the branch xylem sap by diluting the overall ionic content, since atmospheric water is not as ion‐rich as seawater. Moreover, we expected (iii) tides to have a strong effect in modifying xylem sap composition, as high tides would provide roots with an ion‐rich water supply, and thus influence changes throughout the day (i.e., opposite tides will result in different diurnal courses in most xylem sap parameters under study), especially during day time when transpiration rates are high and at night when plants reestablish their internal hydraulic balance.

## 2 Materials and Methods

### 2.1 Plant Material

All measurements were performed on branches collected from two co‐occurring mangrove species: *A. marina* ssp. *australasica* (Walp.) J. Everett and *A. corniculatum* (L.) Blanco. Samples were harvested from mature specimens (at least 3‐m‐tall) growing at the edge of the Hawkesbury River in Brooklyn, New South Wales, Australia (33°54′ S, 151°21′E).

### 2.2 Dynamics in FWU

For testing the dynamics of FWU, we performed two experiments in October 2020. Experiment 1 consisted of submerging and spraying two sets of 1.5‐m‐long excised branches per species, one set collected in late morning (around 11:00 AM) to avoid any dew formation, and the other after dark settled in (around 9:00 PM) to test for the involvement of stomata in the water absorption. At each time point, five branches were collected per species (*n* = 5 per treatment, branches collected from different trees), transported to the lab and dehydrated to a leaf water potential of approximately −4.5 MPa, which is the lowest naturally occurring Ψ measured during a seasonal course survey characterised by favourable conditions (Losso et al. 2023), and it is near the turgor loss point measured in *A. marina* (i.e., −4.9 MPa; Nguyen et al. 2017). After dehydration, branches were allowed to equilibrate in black plastic bags for 30–40 min before starting the experiment. Both experiments were performed in the laboratory under controlled conditions (constant temperature at 21°C). After submerging and spraying treatments, leaf water potential (Ψ) was measured at regular time intervals (i.e., 0, 15, 30, 60, 90, 120, and 150 min). The aim of the first experiment was to test whether leaves were able to take up water and how long it would take to recover leaf Ψ, and check if FWU changes between day and night due to physiological changes (e.g., closed stomata). Excised branches were entirely submerged except for the cut of the stem, which was maintained outside the water, whereas sprayed branches were hung on static supports and sprayed with a spraying bottle every 5–10 min to maintain wetting of the leaf surface. Leaf Ψ was measured on three leaves per branch at each time interval with a Scholander pressure chamber (PMS Instrument Co., Albany, OR, USA). Leaf Ψ was averaged per time interval and species.

**Figure 3:**
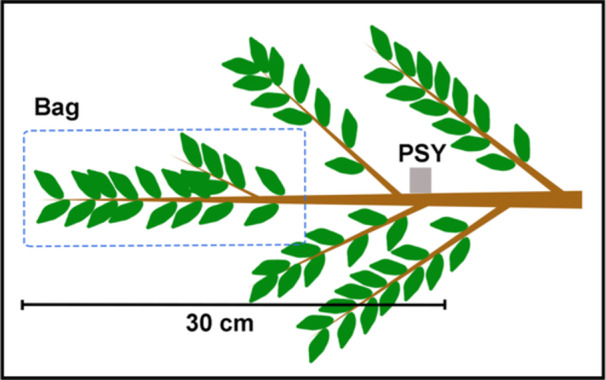
Setup used for testing the dynamics of foliar water uptake (see also Experiment 2 in material and methods). The image indicates the position of the bag used to induce water absorption and the position of the sensor of the psychrometer (PSY). [Color figure can be viewed at wileyonlinelibrary.com]

For Experiment 2, four 1.5‐m‐long excised branches per species (collected from different trees at around 9:00 AM) were allowed to dehydrate to a leaf water potential of about −4.5 and −4.0 MPa for *A. marina* and *A. corniculatum*, respectively. The tip (ca. 20 cm) of each branch was then bagged with a transparent plastic bag (each tip had least 15–20 leaves) and leaves were maintained wet by spraying water into the bag every 10–15 min (see set‐up in Figure 3). A PSY1 Stem Psychrometer sensor coupled with a microvolt data logger used to store data (ICT International, Armidale, NSW, Australia) was installed on the stem at approximately 30 cm from the tip of the branch and stem Ψ was recorded every 10 min. This second experiment was meant to test if water taken up through FWU could move to the stem (i.e., presence of reverse flow) in sufficient amounts to cause an increase in stem water potential. As with Experiment 1, leaf Ψ was measured with a Scholander pressure bomb at different time intervals (i.e., 0, 15, 30, 60, 90, 120, 180, 300 and 420 min; *n* = 2 per branch). Both leaf and stem Ψ were averaged per time interval and species.

### 2.3 Daily Courses in Xylem Sap Composition

For both species under study, xylem sap was extracted and analysed at four‐time points (i.e., predawn, midday, afternoon, and night) across 2 days with contrasting tidal events (Figure 2; time points were independent of tidal events) and characterised by the presence of morning dew on the leaves of trees under study. ﻿ The study site is subject to semi‐diurnal tides that flood the site twice a day, and is characterised by an annual mean precipitation of 1191.3 mm and 158 ± 6 days with morning dew (from data collected at a weather station near the study site; Terrey Hills AWS station number 066059; Bureau of Meteorology). The first day (27.10.2020) was characterised by a first high tide at predawn (1.2 m), which reached its maximum (1.30 m) and minimum (0.50 m) at 7:28 AM and 1:22 PM, respectively (see Figure 2). The following high tide reached 1.40 m at 7:35 PM (Figure 2). The second day (18.11.2020) exhibited opposite tides with the lowest tides recorded at 5:02 AM (0.30 m) and 6:27 PM (0.10 m) and the highest at 11:40 AM (1.70 m) (see Figure 2). At each time point, three 2‐m‐long branches (always from different individuals, even from the previous time points) per species were collected from trees under transpirational conditions, enclosed in plastic bags, and carried to the laboratory for sap extraction. In the laboratory, samples were recut underwater with a fresh razor blade and xylem sap extraction was carried out following (Schenk et al. 2017, 2021). Briefly, the bark was removed from the distal end (ca. 4 cm length) of the main axis to expose the xylem cylinder, which was thoroughly cleaned with deionized water using a high‐pressure dental flosser (WF‐02 Water Flosser, Waterpik Ink, Fort Collins, CO, USA) for 2 min to remove cell debris and cytoplasmic content from the surface. Xylem sap was extracted under vacuum by wrapping half of the exposed xylem cylinder next to the bark with Parafilm (Bemis NA, Neenah, WI, USA), and leaving about 2 cm of xylem cylinder freely exposed. The stem was then inserted into a rubber stopper, avoiding any contact between xylem and stopper, and creating a tight seal between Parafilm and stopper. The exposed xylem cylinder was then cleaned again with deionized water as described above and the excess water was removed with a Kimwipe. Xylem sap was directly collected into 4 mL glass vials, which was embedded in ice. Preliminary experiments indicated visible xylem sap extraction started when the stems were cut to 50–60 cm. Therefore, after a first cut at about 70 cm from the distal end, a series of subsequent cuts (each 2 cm more distal) from the base upwards were performed, which should lead to a release of xylem sap as soon as the largest vessels were cut open. Once dripping sap was observed, further 1 cm cuts were made every minute to allow for a slow and continuous removal of xylem sap. Depending on stem size, 1 to 2 mL of sap were extracted from each branch. Extracted xylem sap samples were frozen until measurements.

For each xylem sap sample, [Na^+^], [Ca^2+^], [K^+^], electrical conductivity (EC) and pH were measured with ion‐selective electrodes (Na‐11, Ca‐11 and K‐11 LAQUAtwin Compact Ion Meter), a conductivity metre (EC‐22 LAQUAtwin Compact Conductivity meter) and a pH metre (pH‐22 Compact pH metre; all from Horiba, Kyoto, Japan). Surface tension (γ) was measured for each sample using a modified device based on the pendant drop technique. The main parts of this device consisted in a syringe (Omnifix‐F 1 mL syringe with a Sterican blunt cannula; B. Braun Melsungen AG, Germany) to allow a controlled production of drops, a camera (Moticam 3 Plus camera, Motic Deutschland GmbH, Wetzlar, Germany) to take photos of the hanging drops, a light source with a diffuser for maintaining standard light conditions for better image analysis, and temperature sensors to account for temperature dependent changes in γ. For each sample, 5–8 drops were generated, and photos acquired. Image analyses were done using the Pendent Drop plug‐in of a Java‐based distribution of ImageJ (US National Institutes of Health, Bethesda, MD, USA; Daerr and Mogne 2016). γ was averaged per sample.

In addition to xylem sap analyses, water potential (Ψ) was measured on at least nine leaves per species (three leaves per sampled branch) at the same time intervals used for xylem sap extraction. Ψ was measured with a Scholander pressure chamber (PMS Instrument Co., Albany, OR, USA) and values were averaged per time interval and species.

### 2.4 Statistics

Differences were tested using one‐way ANOVA followed by Tukey's post hoc comparison for dynamics in FWU (Experiments 1 and 2) and two‐way ANOVA followed by Tukey's post hoc comparison for daily variation in xylem sap parameters. We also assessed the relationships between traits across species and salt management strategies by generating a correlation plot using Pearson's correlation coefficient (xylem sap parameters and Ψ). To obtain species clustering based on traits relationship among species and type of tides, we performed a principal component analysis (PCA) from which an evaluation of differentiation between species and tides events was extracted. All statistical data were analysed with R 3.6.2 (R Core Team 2017) at a probability level of 5%.

## 3 Results

### 3.1 Dynamics in FWU

**Figure 4:**
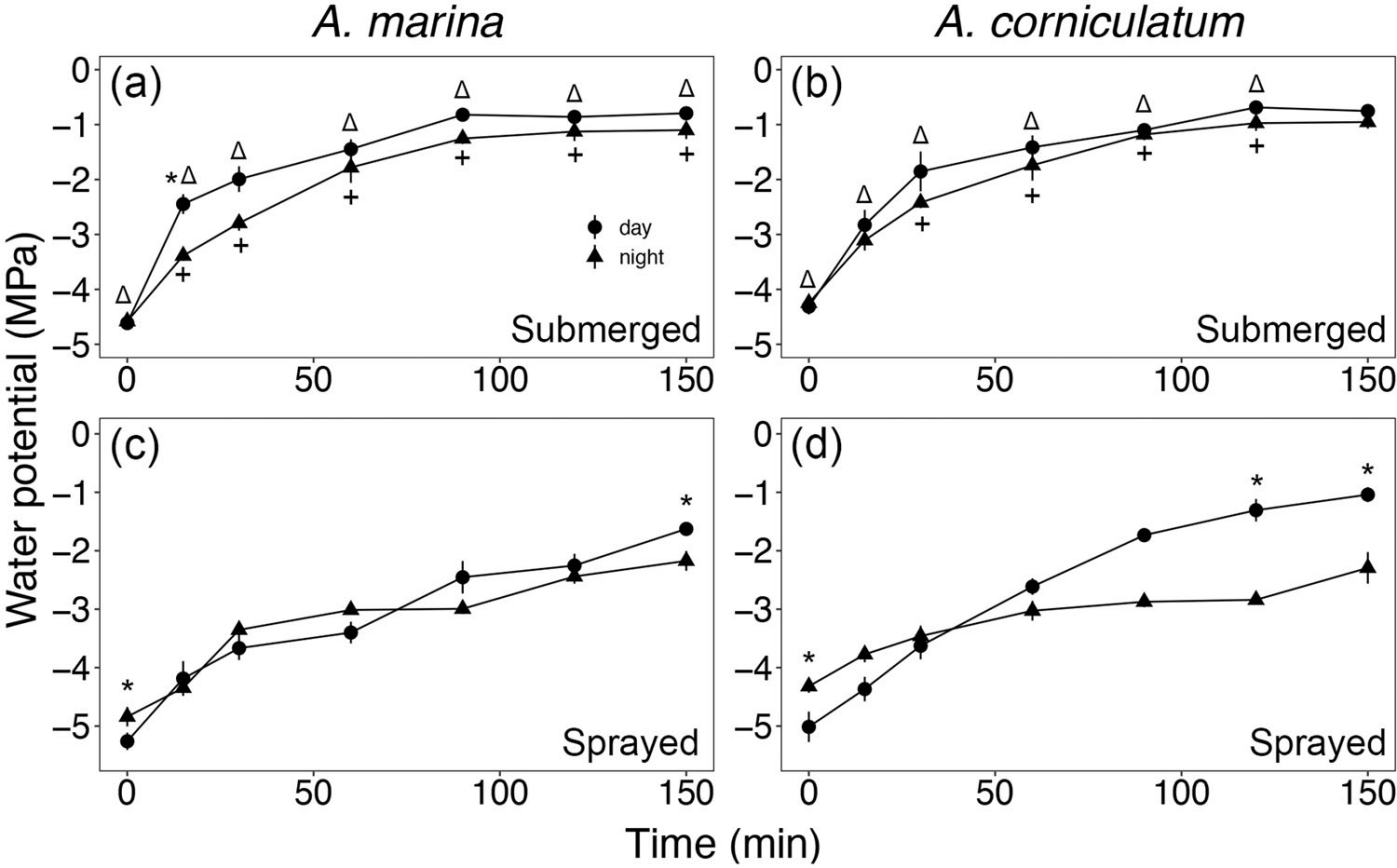
Changes in leaf water potential (MPa) of branches of *Avicennia marina* ssp. *australasica* and *Aegiceris corniculatum* submerged (a and b) and sprayed (c and d) with water both during the day (circles) and night (triangles). Values shown are mean ± SE (*n* = 5). Asterisks indicate differences (*p* < 0.05) between day and night using the same method, triangle indicates differences (*p* < 0.05) between methods during the day (full circles) while plus indicates differences (*p* < 0.05) between methods during the night (full triangles).

**Figure 5:**
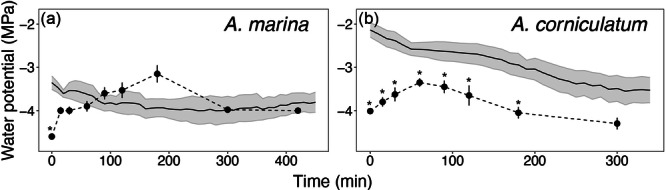
Changes in water potential (MPa) of branches (four per species) of *Avicennia marina* ssp. *australasica* (a) and *Aegiceris corniculatum* (b) over time (min) during exposure to external water (see setup in Figure 3). Circles and dashed lines indicate water potentials (mean ± SE) of leaves (*n* = 4 branches) collected from inside the bag and measured with a Scholander apparatus, whereas the straight line is the average stem water potential measured with the psychrometer delimited by its SE (grey area). Asterisks indicate differences (*p* < 0.05) between methods.

Both experiments testing the dynamics of FWU indicated that mangroves take up water and recover leaf Ψ in very short time intervals (see Figures 4 and 5). Submerged branches exhibited a more pronounced rate of absorption (Figure 4a,b) than sprayed branches (Figure 4c,d), with a drop in leaf Ψ from −4.5 MPa to about −1 and −2 MPa in 150 min in submerged and sprayed, respectively. In submerged branches, absorption was slightly slower at night in both species. No difference between day and night was observed in sprayed branches of *A. marina* (Figure 4c), while night recovery in *A. corniculatum* was not as efficient as day recovery (i.e., lower leaf Ψ at night; Figure 4d).

In Experiment 2, the two species showed a different response (Figure 5). As in Experiment 1, leaf Ψ inside the bag increased and reached its maximum at about −3.2 MPa after 60 and 180 min in *A. marina* and *A. corniculatum*, respectively. After reaching its maximum, leaf Ψ started to decrease again in both species (Figure 5). In *A. marina*, stem Ψ decreased at first and reached more stable values after 200–300 min (ca. −4 MPa) and slowly increased again after about 350 min. The minimum leaf Ψ was reached at the time when stem Ψ started exhibiting stable values (Figure 5a). The subsequent leaf Ψ measured at 300 and 420 min were similar to stem Ψ (Figure 5a). For *A. corniculatum*, stem Ψ was consistently significantly higher than leaf Ψ during the entire experiment, with stem Ψ decreasing from −2.0 MPa to −3.5 MPa and becoming stable after 300 min (Figure 5b).

### 3.2 Daily Courses in Xylem Sap Composition

**Figure 6:**
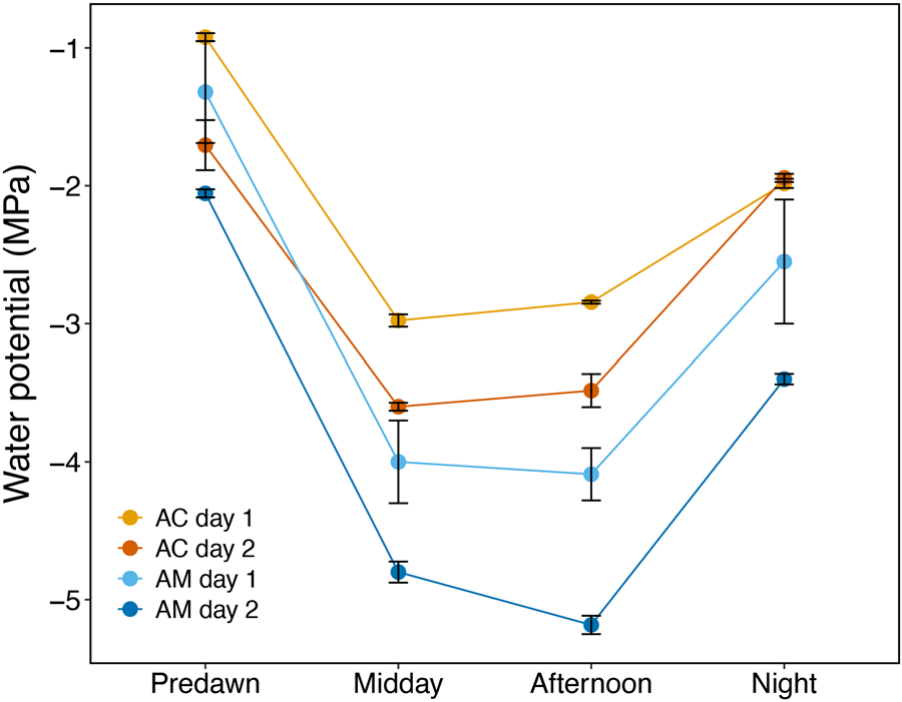
Daily courses of leaf water potential of *Avicennia marina* ssp. *australasica* (AM) and *Aegiceris corniculatum* (AC) measured during 2 days with opposite tides (Days 1 and 2; see also Figure 2c, d). Values shown are mean ± SE (*n* = 3 leaves per sampled branch). Statistical differences are given in Supporting Information S1: Tables S1 and S2. [Color figure can be viewed at wileyonlinelibrary.com]

In both species, leaf water potential (Ψ) showed pronounced daily variation regardless of the tides (Figure 6), with relatively high values at predawn (between −2 and −1 MPa; higher than sea water Ψ) and lower values in the middle of the day (between −5 and −3 MPa during midday and afternoon; Figure 6). Both species then recovered leaf Ψ at night. *A. marina* overall exhibited lower Ψ than *A. corniculatum*, and both species reached lower Ψ during Day 2.

**Figure 7:**
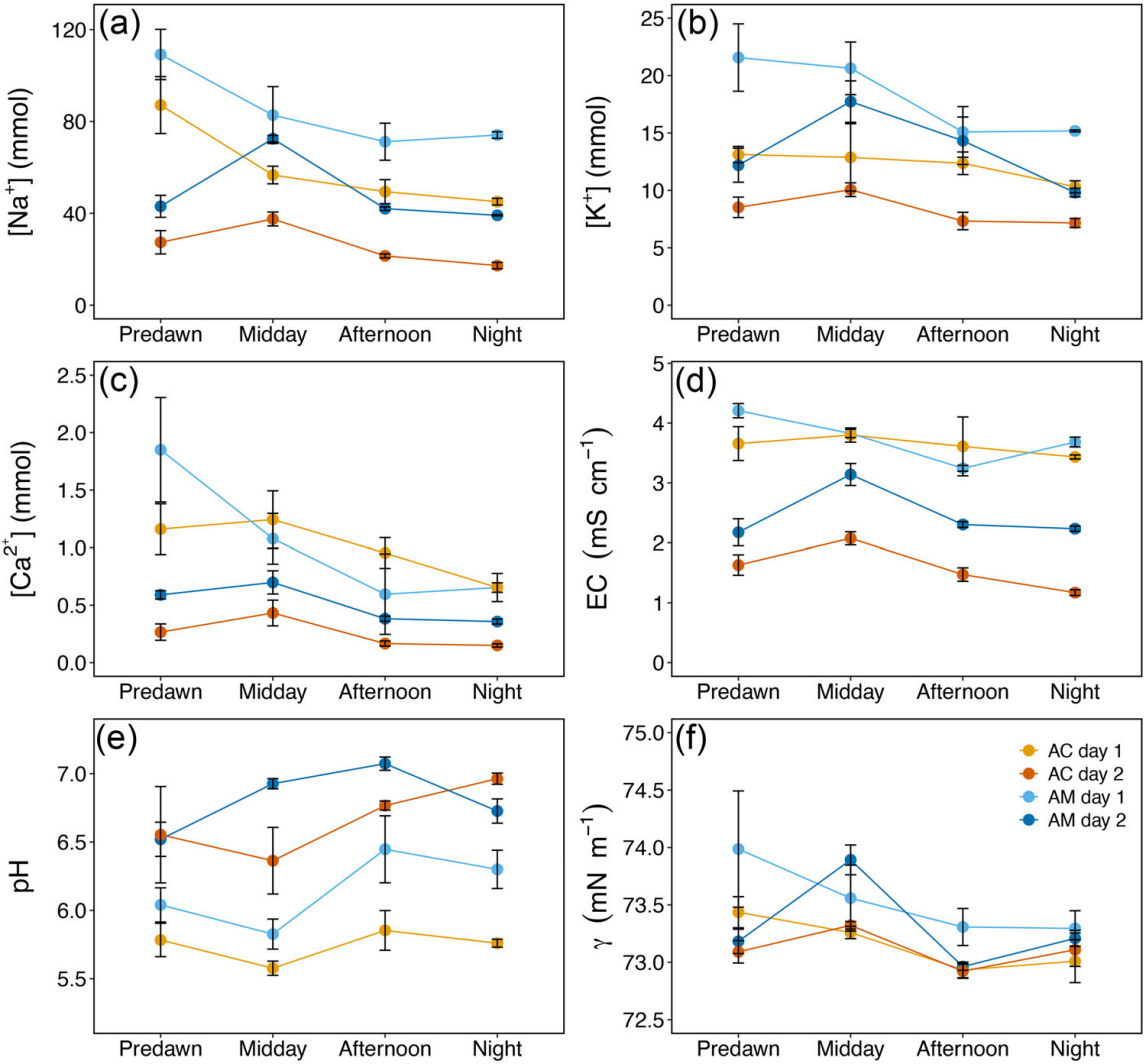
Daily courses of xylem sap sodium ([Na^+^]) (a), potassium ([K^+^]) (b), calcium concentrations ([Ca^2+^]) (c), electrical conductivity (EC) (d), pH (e), and surface tension (γ) (f) of *Avicennia marina* ssp. *australasica* (AM) and *Aegiceris corniculatum* (AC) measured during 2 days with opposite tides (Days 1 and 2; see also Figure 2c,d). Values shown are mean ± SE (*n* = 3 per species and time point). Statistical differences are given in Supporting Information S1: Tables S1 and S2. [Color figure can be viewed at wileyonlinelibrary.com]

Xylem sap analyses indicated species‐specific differences and pronounced daily variation in xylem sap composition during both days under study (Figure 7 and Supporting Information S1: Tables S1 and S2). Daily variation in xylem sap parameters also differed between the two study days, according to tide events. *A. marina* exhibited higher [Na^+^] than *A. corniculatum* (highest values at predawn high tide on Day 1: 109.2 ± 11.0 and 87.1 ± 12.4 mmol in *A. marina* and *A. corniculatum*, respectively; Figure 7a). *A. marina* also exhibited higher [K^+^] and [Ca^2+^] than *A. corniculatum* (highest values at predawn high tide on Day 1: [K^+^] 21.6 ± 3.0 and 13.1 ± 0.7 mmol and [Ca^2+^] 1.8 ± 0.4 and 1.2 ± 0.2 mmol in *A. marina* and *A. corniculatum*, respectively; Figure 7b,c). For both species, EC was overall higher throughout Day 1 than Day 2 (Figure 7d). Consistent with tide times, it peaked at predawn on Day 1 and at midday on Day 2, mirroring changes in ion contents (see Figure 7d).

In both species, xylem sap was more acidic during Day 1 and had more neutral pH values during Day 2 (Figure 7e). In Day 1, both species exhibited the same trend with lower values at predawn and midday, whereas during Day 2 they showed increasing pH already at midday for *A. marina* and in the afternoon for *A. corniculatum* (Figure 7e). For both species, γ was similar to values reported for water (Figure 7f). During Day 1, γ values tended to decrease from predawn to afternoon, while during Day 2 highest values were reported at midday (Figure 7f).

Correlations (based on linear and quadratic relationships) highlighted species‐specific differences in xylem sap parameters in response to tidal changes (Supporting Information S1: Figures S1 and S2). [Na^+^] was the only parameter positively affected by tides in both species in both linear and quadratic relationships (see Supporting Information S1: Figures S1a and Figure S2a). EC and γ were both positively correlated with tides in *A. marina* (Supporting Information S1: Figure S1d,f). In both species, most relationships between xylem sap parameters and tides followed a quadratic function (Supporting Information S1: Figures S1 and S2), except for γ, which showed a linear dependence (Supporting Information S1: Figures S1f and S2f). Overall, the quadratic relationships were more pronounced in *A. corniculatum* than in *A. marina* (see Supporting Information S1: Figure S2). Pearson's correlation coefficients indicated most xylem sap parameters ([Na^+^], [Ca^2+^], [K^+^], EC, pH and γ) to be correlated with each other (see Supporting Information S1: Table S3). Strong positive (> 0.7) correlations were found between [Na^+^] and [K^+^] (0.76), [Na^+^] and EC (0.85), and [Ca^2+^] and EC (0.7), while strong negative (< −0.7) correlations were found between [Ca^2+^] and pH (−0.8), pH and EC (−0.7). Changes in xylem sap composition were not related to Ψ.

**Figure 8:**
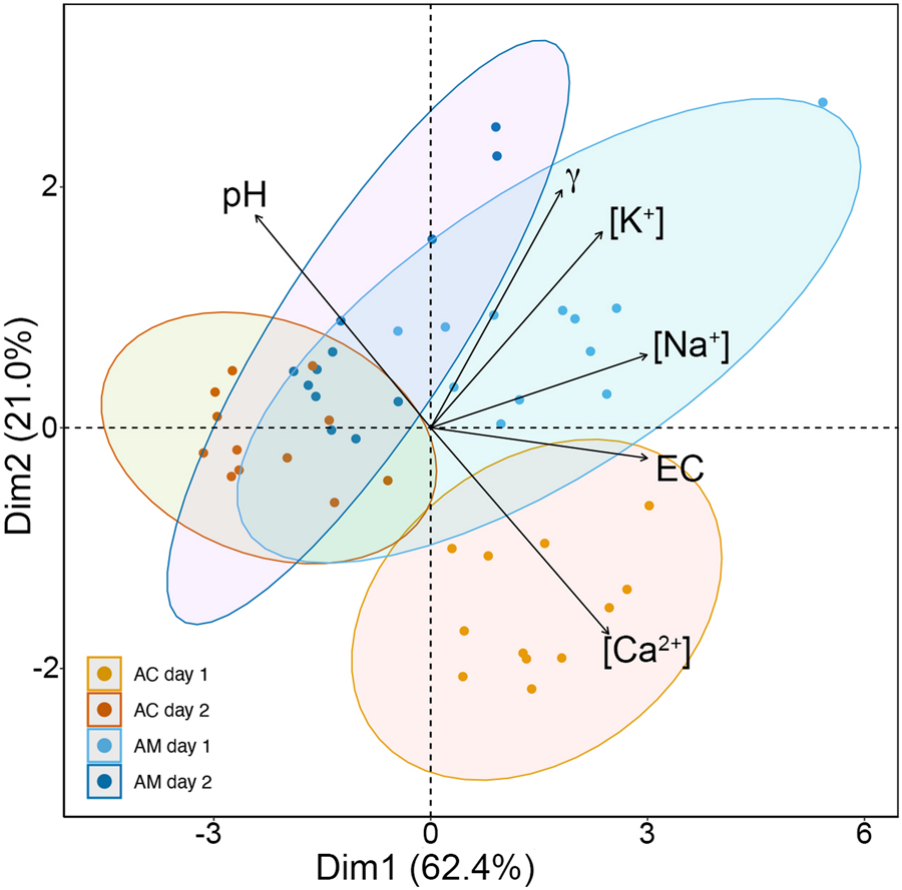
Principal component analysis (PCA) showing the general associations among xylem sap parameters ([Na^+^], [K^+^], [Ca^2+^], electrical conductivity (EC), pH and surface tension (γ)) of *Avicennia marina* ssp. *australasica* (AM) and *Aegiceris corniculatum* (AC) during two contrasting tides events (Days 1 and 2; see also Figure 2c,d). Arrows indicate the contributions of the variables to the principal component axis. Circles represent clusters grouping the two species in depending on the tides event. [Color figure can be viewed at wileyonlinelibrary.com]

The PCA analysis (Figure 8) extracted three principal components axis (PC1, PC2 and PC3) with eigenvalues higher than 0.5 (only two axes with eigenvalues > 0.8), explaining a cumulative variance of 92.0% for six traits (see Supporting Information S1: Table S4). PC1 explained 62.4% of the variance and had a positive loading for [Na^+^], EC and [K^+^], while PC2 explained 21.0% of the variance and had a positive loading for pH and γ and a negative loading for [Ca^2+^]. The PCA showed very clear clusters indicating xylem sap composition to differ between the two species as well as within species between different tides dynamics (Figure 8).

## 4 Discussion

In this study, we provided evidence that two co‐occurring salt‐secreting mangroves can absorb water via leaves leading to recovery of leaf water potential in relatively short time frames. Both species exhibited pronounced daily variation in their xylem sap composition. Changes in [Na^+^] were clearly related to tides, whereas variation in most of the other xylem sap parameters were likely to be driven by transpiration. We also show that the presence of dew on leaves at predawn may have acted as external water source, buffering leaf Ψ locally but not affecting xylem sap composition at a systemic level.

Observed recovery in leaf Ψ after spraying/submersion (Figures 4 and 5), as well as high predawn leaf Ψ measured with the presence of morning dew (Figure 6), were consistent with other studies indicating that an alternative water supply like FWU is likely required for mangroves to achieve full turgor (Reef et al. 2015; Nguyen et al. 2017; Schreel et al. 2019) and support xylem embolism removal (Fuenzalida et al. 2023). Accordingly, Fuenzalida et al. (2019) reported leaves to be the dominant organs for external water absorption in *A. marina*. However, from our experiments it is hard to differentiate whether water was absorbed exclusively by leaves or also by the bark (Earles et al. 2016; Losso et al. 2021); bark might have played a major role in water absorption when branches were submerged, as the recovery in leaf Ψ was significantly more efficient than in sprayed branches (Figure 4). This finding is consistent with a recent study (Beckett et al. 2024), which suggested that bark water uptake through lenticels in stems of *A. marina* may play a crucial role in modulating xylem Ψ and maintaining living tissue hydration. Under the same laboratory conditions, the time of the day (day vs. night) did not influence water absorption (Figure 4), which might indicate that the involvement of stomata in the water absorption is not relevant and/or that they stay open overnight. Beckett et al. (2024) also found no differences in bark water uptake between dark and light conditions. Under field conditions, FWU from dew mainly occur later in the night simply because favourable conditions that support condensation were reached at this time point.

Foliar water uptake (FWU) experiments indicated that both species were able to recover leaf Ψ in relatively short and similar time periods (recovery of ca. 2.5 MPa in less than 3 h; Figure 4, see also Figure 1), and agree with Schreel et al. (2019), who reported *A. marina* seedlings to recover leaf Ψ from −3.5 to ﻿−1.7 MPa in less than 3 h of continuous water sprayings. Other studies observed slower recovery in *A. marina* (recovery of ca. 1–2 MPa in 4 h; Fuenzalida et al. 2019) using similar leaf‐wetting techniques. This is most likely an environmental adaptation as samples used by Schreel et al. (2019) were collected very close to our sampling site, whereas the mangroves used by Fuenzalida et al. (2019) were growing in tropical climate at much lower latitudes, and belong to a different subspecies (i.e., *eucalyptifolia*) with differences in leaf morphology and anatomy. Results thus indicate mangroves growing at higher latitudes to repair leaf Ψ more efficiently when wetting events occur. Interestingly, in Experiment 2 (Figure 5), leaf Ψ inside the bags also increased during the first hours but then dropped again. Leaf Ψ recovery was slightly more pronounces in *A. marina*. Stem Ψ showed a different pattern as it slowly became more negative until it reached stable values after about 4–5 h (Figure 5). Leaf FWU thus obviously allowed an increase in Ψ of sprayed leaves. The consecutive drop in leaf Ψ as well as the lack of a (transient) increase in Ψ of basal stem parts was probably due to parallel transpirational water losses over unsprayed and unbagged leaves. It is also worth noting that leaf and stem Ψ were measured with two different methods, and absolute Ψ values might thus differ. Further experiments with various portions of sprayed versus unsprayed leaves (including unsprayed controls), as well as sap flow measurements, would be needed for insight into FWU, evidence for reverse flow, and transpiration dynamics. We suggest that *in natura*, wetting of entire branches and plants may lead to overall increases of Ψ. From previous studies, the more salt‐tolerant *A. marina* is known to be able to repair xylem embolism via FWU, but only over longer time scales (> 10 h; Fuenzalida et al. 2023) than those used in our study (< 6–7 h). According to previous studies, only either prolonged rainfall events or a relative humidity of 80%–95% can trigger a complete reversal of the flow (Steppe et al. 2018; Schreel et al. 2019; Coopman et al. 2021).

Leaf Ψ followed classic diurnal patterns of morning decline and evening rise, with lower values measured in the middle of the day (Figure 6), when transpiration rates are expected to be highest. Similar patterns have already been observed in other mangroves (Vandegehuchte et al. 2014), and are consistent with previously observed diurnal transpiration courses (López‐Portillo et al. 2014). As expected, when dew was present on mangrove leaves, predawn leaf Ψ was much higher (between −2 and −1 MPa; Figure 6) than expected for plants growing under saline conditions, suggesting that the use of dew for local Ψ recovery is a common process in both mangrove species under study. Leaf Ψ never reached critical levels (see Gauthey et al. 2022), suggesting that occasional FWU uptake of dew helps these two species to maintain leaf Ψ within their safety margins. During Day 2, leaf Ψ was overall lower, which could have been caused by the higher temperatures (Figure 2b) and/or higher concentrations of ions potentially leading to imbalances in the osmotic potential of living tissues. It also should be noted that the 2 days under study were preceded by 2 days of contrasting conditions, with only the day before Day 1 having some rainfall (82.2 mm). However, we can assume that the rain had no effect on our measurements, as the patterns in leaf Ψ were very similar on both days.

Clusters of measured xylem sap parameters defined minor differences between species and identified a partial effect of tide events (Figure 8). Tides influenced changes in [Na^+^] (Supporting Information S1: Figures S1 and S2), which is the dominant ion in the xylem sap of salt‐secreting mangroves (see also Krishnamurthy et al. 2014; López‐Portillo et al. 2014) and was more abundant in the more salt‐tolerant *A. marina*. In previous studies, authors observed salt‐secreting halophytes to reach their highest xylem sap salt concentrations around midday, as water uptake and high transpiration during the day would overwhelm salt secretion rates (Scholander et al. 1962; Drennan & Pammenter 1982; Sobrado 2002). Similarly, López‐Portillo *et al*. (2005) reported xylem sap osmotic potential of *Avicennia germinans* to be more negative at midday than at predawn, whereas no difference was observed in the xylem sap of the non‐salt secreting *Conocarpus ﻿erectus*. In a study on ﻿*Laguncularia racemosa* (non‐salt secreting; Sobrado 2004), changes in [Na^+^] explained about 51%–58% of increase in xylem osmolality under both field and glasshouse conditions. In our study, xylem sap [Na^+^] showed different daily patterns depending on the tide dynamics, which were consistent in both mangrove species (Figure 7a; see also Supporting Information S1: Figures S1 and S2).

Pronounced daily changes were also observed in the other analysed xylem sap parameters (see Figure 7). Changes in [K^+^] were reflected by changes in [Na^+^] (Figures 7a,b and 8). K^+^ is a crucial ion for photosynthetic and respiration processes (Ball 1988), protein synthesis (Peoples and Koch 1979), and plays a central role in enzymatic actions and maintaining the osmotic balance and electrical regulation (Coskun et al. 2013), which help supporting salt tolerance (Rains and Epstein 1967; Parida, Das, and Mittra 2004). The uptake of K^+^ and Na^+^ occurs through the same channels in roots (Cramer 2002). These channels are supposedly more selective for K^+^ than for Na^+^, but, as sea water [Na^+^] is considerably higher than [K^+^], higher xylem sap [Na^+^] is expected. K^+^ and Na^+^ can also be used by mangroves for ion‐mediated regulation of xylem hydraulic conductivity (the so‐called “ionic effect”; e.g., López‐Portillo*et al*. 2005; Nardini, Salleo, and Jansen 2011), and it has been speculated that use of these ions may be related to their availability in the environment in which the plants grow (López‐Portillo et al. 2014). Accordingly, both [Na^+^] and [K^+^] reflected tidal changes, with high tides providing higher xylem sap concentrations of both ions (see Figures 2c,d and 7a,b). However, changes in [K^+^] may not be strictly related to tidal variation, as plants can recirculate K^+^ within the xylem and phloem loop and thus maintain required levels of hydraulic conductance via the ionic effect (e.g., Nardini, Salleo, and Jansen 2011).

Xylem sap pH fell within the range reported for other land species (4.5–7.4; Teskey et al. 2008; Losso et al. 2018), and all values were lower than sea water pH (7.7) with an overall trend towards more acidic values during Day 1 in both species and towards less acidic values during the high tide of Day 2 (i.e., midday and afternoon; Figure 7e). Both xylem sap acidification (Sharp and Davies 2009; Losso et al. 2018; Pagliarani et al. 2019) and alkalinization (Wilkinson and Davies 1997, 2008; Beis, Zotos, and Patakas 2009; Salomón et al. 2016) have been reported to correspond to drought stress, depending on species and conditions. Acidification has been shown to increase acidic invertase activity and sucrose hydrolysis, which would reduce the xylem sap osmotic potential (Secchi and Zwieniecki 2016; Tomasella et al. 2021) in response to drought. This may explain the generally low pH found in the mangroves under study. However, alkalinization has been shown to act as a stress signal, leading to increased abscisic acid concentrations and consequently to leaf stomatal closure, thereby reducing water loss under drought. This may have occurred during daytime (especially on the warmer Day 2), although further biochemical studies (on days with different stress intensities) would be required to disentangle these processes and the functional role of the observed pH changes in mangroves. Daily variation in pH were negatively related to daily variation in [Ca^2+^] (Figure 8 and Supporting Information S1: Table S3), which is very interesting as both parameters are connected to drought and salinity stress responses (Kader and Lindberg 2010). Together with Ca^2+^, protons also function as second messengers in plant cells during and upon salinity stress (e.g., Roos 2000; Roos et al. 2006; Kader and Lindberg 2010). Under salt stress, Ca^2+^ is known for being an important messenger (Hadi and Karimi 2012), to preserve the structural integrity of cell membranes (Miyama and Tada 2008) and have beneficial effects on the overall ionic homoeostasis (Lu et al. 2013; Lang et al. 2014). At the cytosolic level, changes in [Ca^2+^] are reported within seconds after sensing salinity stress (Kader and Lindberg 2010). In our study, xylem sap [Ca^2+^] was highest in the morning and at midday during Days 1 and 2, respectively (Figure 7c). Observed peaks corresponded to high tides (Figure 2c,d), increased [Na^+^] (Figure 7a), and thus increased salinity stress, and therefore Ca^2+^ could act as a signal against salt stress.

Our study provides new insights into the recovery times of leaf Ψ of two mangrove species via FWU upon exposure to atmospheric water. These results support recent assertions (Steppe et al. 2018; Schreel, Van de Wal, et al. 2019; Coopman et al. 2021) on the importance of leaf‐wetting events in the maintenance of leaf gas exchange and plant productivity, which may affect plant fitness under changing climatic scenarios. This is particularly true in Australia, where mangrove ecosystems are predicted to be subjected to increasing temperatures and decreasing precipitations (IPCC 2014).﻿﻿ Dew was present on leaves during both daily courses and predawn leaf Ψ indicated that mangroves might have taken advantage of this atmospheric phenomenon for local hydraulic adjustments. Whereas dew played an important role in localized daily water adjustments, it did not affect the composition of mangrove xylem sap at a systemic level, which exhibited pronounced and independent daily variations. Hence, FWU from dew may not be sufficient to help compensate for future higher drought intensities as larger water sources (or high relative humidity) are required over longer time frames.

## Supporting information

Supporting information.

## Data Availability

The data supporting the findings of this study are available from the corresponding author on reasonable request.
