# Supplementary material for: Foliar Water Uptake Supports Water Potential Recovery but Does Not Affect Xylem Sap Composition in Two Salt‐Secreting Mangroves
Source: Plant Cell Environ. 2024 Dec 16;48(5):3027–37. doi: 10.1111/pce.15332 (PMC11963484; doi:10.1111/pce.15332)

**Table S1.** F and p value of the two-way ANOVA of leaf water potential and xylem sap parameters measured on *Avicennia marina* (AM) and *Aegiceras corniculatum* (AC) during day 1 and day 2. Significant p values (<0.05) are shown in bold.

|  | **Leaf water potential (Ψ)** | | **Sodium (Na^+^)** | | **Potassium (K^+^)** | | **Calcium (Ca^2+^)** | | **Electrical conductivity (EC)** | | **pH** | | **Surface tension (γ)** | |
| --- | --- | --- | --- | --- | --- | --- | --- | --- | --- | --- | --- | --- | --- | --- |
| **Species** | **F value** | **p value** | **F value** | **p value** | **F value** | **p value** | **F value** | **p value** | **F value** | **p value** | **F value** | **p value** | **F value** | **p value** |
| AM | 25.02 | **0.00013** | 61.87 | **6.90E-07** | 11.872 | **0.00332** | 11.798 | **0.0034** | 223.4 | **8.05E-11** | 51.51 | **2.20E-06** | 1.926 | 0.1842 |
|  | 31.32 | **5.60E-08** | 8.02 | **0.00041** | 3.269 | **0.02711** | 3.701 | **0.0169** | 12.5 | **2.93E-05** | 3.973 | **0.0126** | 2.523 | 0.0651 |
| AC | 75.49 | **1.87E-07** | 129.749 | **4.36E-09** | 19.958 | **0.000389** | 605.171 | **3.85E-14** | 169.045 | **6.39E-10** | 59.685 | **8.70E-07** | 0.39 | 0.5413 |
|  | 142.36 | **5.69E-13** | 7.442 | **0.000619** | 1.123 | 0.392846 | 9.327 | **0.000173** | 1.679 | 0.19 | 1.442 | 0.259 | 3.336 | **0.0251** |
| AM *versus* AC | 118 | **2.89E-12** | 43.62 | **1.91E-07** | 49.149 | **6.04E-08** | 59.12 | **9.16E-09** | 30.8 | **4.01E-06** | 13.91 | **0.000744** | 10.278 | **0.00305** |
|  | 40.6 | **2.32E-16** | 19.31 | **9.85E-12** | 4.293 | **0.000317** | 31.07 | **1.18E-14** | 29.49 | **2.51E-14** | 10.14 | **4.13E-08** | 2.526 | **0.01488** |

**Table S2.** Results of the two-way ANOVA followed by Tukey’s post hoc comparison. $Time indicates the time of the day (i.e., Predawn, Midday, Afternoon or Night) at which leaf water potential and xylem sap parameters were measured on *Avicennia marina* (AM) and *Aegiceras corniculatum* (AC) during day 1 and day 2. *diff* is the difference between means of the two groups, *lwr* and *upr* are the lower and the upper endpoints of the 95% confidence interval, respectively, and *p adj* is the p-value after adjustment for the multiple comparisons. Significant *p adj* (<0.05) are shown in bold.

|  | |  | | **Leaf water potential (Ψ)** | | | | | | | | **Sodium (Na^+^)** | | | | | | | | **Potassium (K^+^)** | | | | | | | | **Calcium (Ca^2+^)** | | | |
| --- | --- | --- | --- | --- | --- | --- | --- | --- | --- | --- | --- | --- | --- | --- | --- | --- | --- | --- | --- | --- | --- | --- | --- | --- | --- | --- | --- | --- | --- | --- | --- |
| **$Species** | | **$Time** | | **diff** | | **lwr** | | **upr** | | **p adj** | | **diff** | | **lwr** | | **upr** | | **p adj** | | **diff** | | **lwr** | | **upr** | | **p adj** | | **diff** | **lwr** | **upr** | **p adj** |
| AM | | Midday1-Predawn1 | | -2.678 | | -3.879 | | -1.476 | | **0.000** | | -36.397 | | -69.525 | | -3.270 | | **0.026** | | -0.938 | | -10.190 | | 8.314 | | 1.000 | | -0.773 | -1.856 | 0.309 | 0.273 |
|  | | Afternoon1-Predawn1 | | -2.772 | | -3.974 | | -1.571 | | **0.000** | | -47.997 | | -81.124 | | -14.870 | | **0.002** | | -6.479 | | -15.731 | | 2.773 | | 0.293 | | -1.255 | -2.338 | -0.172 | **0.017** |
|  | | Night1-Predawn1 | | -1.228 | | -2.429 | | -0.026 | | **0.043** | | -45.097 | | -78.224 | | -11.970 | | **0.004** | | -6.394 | | -15.646 | | 2.858 | | 0.307 | | -1.197 | -2.280 | -0.114 | **0.025** |
|  | | Predawn2-Predawn1 | | 0.135 | | -1.067 | | 1.336 | | 1.000 | | -38.499 | | -71.626 | | -5.372 | | **0.017** | | -4.774 | | -14.026 | | 4.478 | | 0.637 | | -0.723 | -1.806 | 0.360 | 0.345 |
|  | | Afternoon1-Midday1 | | -0.094 | | -1.296 | | 1.107 | | 1.000 | | -11.599 | | -44.727 | | 21.528 | | 0.917 | | -5.542 | | -14.794 | | 3.710 | | 0.469 | | -0.481 | -1.564 | 0.602 | 0.777 |
|  | | Night1-Midday1 | | 1.450 | | 0.248 | | 2.652 | | **0.013** | | -8.700 | | -41.827 | | 24.428 | | 0.981 | | -5.456 | | -14.708 | | 3.796 | | 0.487 | | -0.423 | -1.506 | 0.660 | 0.865 |
|  | | Midday2-Midday1 | | 0.068 | | -1.134 | | 1.270 | | 1.000 | | 27.332 | | -5.795 | | 60.459 | | 0.148 | | 1.705 | | -7.547 | | 10.957 | | 0.998 | | 0.159 | -0.924 | 1.241 | 0.999 |
|  | | Night1-Afternoon1 | | 1.544 | | 0.343 | | 2.746 | | **0.007** | | 2.900 | | -30.227 | | 36.027 | | 1.000 | | 0.085 | | -9.167 | | 9.337 | | 1.000 | | 0.058 | -1.025 | 1.141 | 1.000 |
|  | | Afternoon2-Afternoon1 | | -0.221 | | -1.422 | | 0.981 | | 0.998 | | 8.483 | | -24.644 | | 41.610 | | 0.983 | | 3.836 | | -5.415 | | 13.088 | | 0.828 | | 0.324 | -0.759 | 1.407 | 0.961 |
|  | | Night2-Night1 | | 0.018 | | -1.184 | | 1.220 | | 1.000 | | 2.683 | | -30.444 | | 35.811 | | 1.000 | | -0.767 | | -10.019 | | 8.485 | | 1.000 | | 0.241 | -0.842 | 1.324 | 0.993 |
|  | | Midday2-Predawn2 | | -2.744 | | -3.946 | | -1.543 | | **0.000** | | 29.433 | | -3.694 | | 62.561 | | 0.101 | | 5.542 | | -3.710 | | 14.794 | | 0.469 | | 0.108 | -0.975 | 1.191 | 1.000 |
|  | | Afternoon2-Predawn2 | | -3.128 | | -4.329 | | -1.926 | | **0.000** | | -1.015 | | -34.142 | | 32.112 | | 1.000 | | 2.131 | | -7.121 | | 11.383 | | 0.991 | | -0.208 | -1.291 | 0.875 | 0.997 |
|  | | Night2-Predawn2 | | -1.344 | | -2.546 | | -0.143 | | **0.023** | | -3.915 | | -37.042 | | 29.212 | | 1.000 | | -2.387 | | -11.639 | | 6.865 | | 0.983 | | -0.233 | -1.316 | 0.850 | 0.994 |
|  | | Afternoon2-Midday2 | | -0.383 | | -1.585 | | 0.818 | | 0.947 | | -30.448 | | -63.575 | | 2.679 | | 0.084 | | -3.410 | | -12.662 | | 5.842 | | 0.895 | | -0.316 | -1.399 | 0.767 | 0.966 |
|  | | Night2-Midday2 | | 1.400 | | 0.198 | | 2.602 | | **0.017** | | -33.348 | | -66.475 | | -0.221 | | **0.048** | | -7.929 | | -17.181 | | 1.323 | | 0.123 | | -0.341 | -1.424 | 0.742 | 0.950 |
|  | | Night2-Afternoon2 | | 1.783 | | 0.582 | | 2.985 | | **0.002** | | -2.900 | | -36.027 | | 30.227 | | 1.000 | | -4.519 | | -13.770 | | 4.733 | | 0.693 | | -0.025 | -1.108 | 1.058 | 1.000 |
| AC | | Midday1-Predawn1 | | -2.056 | | -2.455 | | -1.656 | | **0.000** | | -30.448 | | -57.000 | | -3.896 | | **0.019** | | -0.256 | | -6.301 | | 5.790 | | 1.000 | | 0.083 | -0.613 | 0.780 | 1.000 |
|  | | Afternoon1-Predawn1 | | -1.922 | | -2.322 | | -1.523 | | **0.000** | | -37.698 | | -64.250 | | -11.146 | | **0.003** | | -0.767 | | -6.813 | | 5.278 | | 1.000 | | -0.607 | -1.304 | 0.089 | 0.112 |
|  | | Night1-Predawn1 | | -1.061 | | -1.460 | | -0.662 | | **0.000** | | -42.048 | | -68.600 | | -15.496 | | **0.001** | | -2.813 | | -8.859 | | 3.232 | | 0.738 | | -1.206 | -1.902 | -0.510 | **0.000** |
|  | | Predawn2-Predawn1 | | -0.282 | | -0.682 | | 0.117 | | 0.284 | | -26.062 | | -52.614 | | 0.490 | | 0.056 | | -0.703 | | -6.749 | | 5.342 | | 1.000 | | -0.420 | -1.116 | 0.276 | 0.461 |
|  | | Afternoon1-Midday1 | | 0.133 | | -0.266 | | 0.533 | | 0.933 | | -7.250 | | -33.801 | | 19.302 | | 0.976 | | -0.512 | | -6.557 | | 5.534 | | 1.000 | | -0.690 | -1.387 | 0.006 | 0.053 |
|  | | Night1-Midday1 | | 0.994 | | 0.595 | | 1.394 | | **0.000** | | -11.599 | | -38.151 | | 14.952 | | 0.791 | | -2.558 | | -8.603 | | 3.488 | | 0.814 | | -1.289 | -1.986 | -0.593 | **0.000** |
|  | | Midday2-Midday1 | | -0.121 | | -0.520 | | 0.278 | | 0.959 | | 14.535 | | -12.016 | | 41.087 | | 0.572 | | 1.087 | | -4.959 | | 7.133 | | 0.998 | | -0.337 | -1.033 | 0.360 | 0.702 |
|  | | Night1-Afternoon1 | | 0.861 | | 0.462 | | 1.260 | | **0.000** | | -4.350 | | -30.902 | | 22.202 | | 0.999 | | -2.046 | | -8.092 | | 3.999 | | 0.929 | | -0.599 | -1.295 | 0.098 | 0.120 |
|  | | Afternoon2-Afternoon1 | | -0.138 | | -0.537 | | 0.262 | | 0.922 | | 5.691 | | -20.861 | | 32.243 | | 0.994 | | -1.130 | | -7.175 | | 4.916 | | 0.997 | | 0.087 | -0.609 | 0.784 | 1.000 |
|  | | Night2-Night1 | | 0.541 | | 0.142 | | 0.940 | | **0.005** | | 5.836 | | -20.716 | | 32.388 | | 0.993 | | 0.746 | | -5.300 | | 6.791 | | 1.000 | | 0.670 | -0.027 | 1.366 | 0.064 |
|  | | Midday2-Predawn2 | | -1.894 | | -2.294 | | -1.495 | | **0.000** | | 10.149 | | -16.402 | | 36.701 | | 0.877 | | 1.535 | | -4.511 | | 7.580 | | 0.984 | | 0.166 | -0.530 | 0.863 | 0.989 |
|  | | Afternoon2-Predawn2 | | -1.778 | | -2.177 | | -1.378 | | **0.000** | | -5.945 | | -32.497 | | 20.607 | | 0.992 | | -1.194 | | -7.239 | | 4.852 | | 0.996 | | -0.100 | -0.796 | 0.597 | 1.000 |
|  | | Night2-Predawn2 | | -0.238 | | -0.637 | | 0.162 | | 0.476 | | -10.149 | | -36.701 | | 16.402 | | 0.877 | | -1.364 | | -7.410 | | 4.681 | | 0.992 | | -0.116 | -0.813 | 0.580 | 0.999 |
|  | | Afternoon2-Midday2 | | 0.117 | | -0.283 | | 0.516 | | 0.966 | | -16.094 | | -42.646 | | 10.458 | | 0.455 | | -2.728 | | -8.774 | | 3.317 | | 0.765 | | -0.266 | -0.963 | 0.430 | 0.877 |
|  | | Night2-Midday2 | | 1.657 | | 1.257 | | 2.056 | | **0.000** | | -20.299 | | -46.851 | | 6.253 | | 0.209 | | -2.899 | | -8.944 | | 3.147 | | 0.710 | | -0.283 | -0.979 | 0.414 | 0.842 |
|  | | Night2-Afternoon2 | | 1.540 | | 1.141 | | 1.939 | | **0.000** | | -4.205 | | -30.757 | | 22.347 | | 0.999 | | -0.171 | | -6.216 | | 5.875 | | 1.000 | | -0.017 | -0.713 | 0.680 | 1.000 |
| AM *versus* AC | | ACPredawn1-AMPredawn1 | | -0.593 | | -1.552 | | 0.366 | | 0.630 | | -1.800 | | -33.953 | | 30.352 | | 1.000 | | -2.845 | | -11.215 | | 5.525 | | 0.995 | | 0.595 | -0.380 | 1.570 | 0.651 |
|  | | ACMidday1-AMMidday1 | | 0.029 | | -0.930 | | 0.988 | | 1.000 | | 4.149 | | -28.004 | | 36.301 | | 1.000 | | -2.163 | | -10.533 | | 6.207 | | 1.000 | | 1.451 | 0.476 | 2.427 | **0.000** |
|  | | ACAfternoon1-AMAfternoon1 | | 0.257 | | -0.702 | | 1.216 | | 1.000 | | 8.498 | | -23.654 | | 40.651 | | 1.000 | | 2.867 | | -5.503 | | 11.237 | | 0.994 | | 1.243 | 0.267 | 2.218 | **0.004** |
|  | | ACNight1-AMNight1 | | -0.427 | | -1.386 | | 0.532 | | 0.945 | | 1.249 | | -30.904 | | 33.401 | | 1.000 | | 0.735 | | -7.635 | | 9.105 | | 1.000 | | 0.585 | -0.390 | 1.561 | 0.674 |
|  | | ACPredawn2-AMPredawn2 | | -0.643 | | -1.602 | | 0.316 | | 0.504 | | 4.588 | | -27.564 | | 36.741 | | 1.000 | | 1.929 | | -6.441 | | 10.299 | | 1.000 | | -1.039 | -2.014 | -0.064 | **0.028** |
|  | | ACMidday2-AMMidday2 | | 0.207 | | -0.752 | | 1.166 | | 1.000 | | -14.696 | | -46.848 | | 17.457 | | 0.933 | | -2.078 | | -10.448 | | 6.292 | | 1.000 | | -0.981 | -1.956 | -0.006 | **0.047** |
|  | | ACAfternoon2-AMAfternoon2 | | 0.707 | | -0.252 | | 1.666 | | 0.354 | | -0.342 | | -32.494 | | 31.811 | | 1.000 | | -1.396 | | -9.766 | | 6.974 | | 1.000 | | -0.931 | -1.906 | 0.044 | 0.074 |
|  | | ACNight2-AMNight2 | | 0.463 | | -0.496 | | 1.422 | | 0.902 | | -1.647 | | -33.799 | | 30.506 | | 1.000 | | 2.952 | | -5.418 | | 11.322 | | 0.993 | | -0.923 | -1.898 | 0.052 | 0.079 |
|  |  | | **Electrical conductivity (EC)** | | | | | | | | **pH** | | | | | | | | **Surface tension (γ)** | | | | | | | |  |  |  |  |  |
| **$Species** | **$Time** | | **diff** | | **lwr** | | **upr** | | **p adj** | | **diff** | | **lwr** | | **upr** | | **p adj** | | **diff** | | **lwr** | | **upr** | | **p adj** | |  |  |  |  |  |
| AM | Midday1-Predawn1 | | -0.380 | | -0.972 | | 0.212 | | 0.388 | | -0.213 | | -0.848 | | 0.422 | | 0.931 | | -0.427 | | -1.553 | | 0.698 | | 0.880 | |  |  |  |  |  |
|  | Afternoon1-Predawn1 | | -0.963 | | -1.555 | | -0.372 | | **0.001** | | 0.407 | | -0.228 | | 1.042 | | 0.392 | | -0.678 | | -1.804 | | 0.447 | | 0.461 | |  |  |  |  |  |
|  | Night1-Predawn1 | | -0.523 | | -1.115 | | 0.068 | | 0.104 | | 0.260 | | -0.375 | | 0.895 | | 0.837 | | -0.691 | | -1.816 | | 0.434 | | 0.440 | |  |  |  |  |  |
|  | Predawn2-Predawn1 | | -0.755 | | -1.346 | | -0.163 | | **0.008** | | -0.178 | | -0.813 | | 0.457 | | 0.972 | | -0.578 | | -1.703 | | 0.547 | | 0.641 | |  |  |  |  |  |
|  | Afternoon1-Midday1 | | -0.583 | | -1.175 | | 0.008 | | 0.055 | | 0.620 | | -0.015 | | 1.255 | | 0.058 | | -0.251 | | -1.376 | | 0.874 | | 0.992 | |  |  |  |  |  |
|  | Night1-Midday1 | | -0.143 | | -0.735 | | 0.448 | | 0.988 | | 0.473 | | -0.162 | | 1.108 | | 0.232 | | -0.264 | | -1.389 | | 0.862 | | 0.990 | |  |  |  |  |  |
|  | Midday2-Midday1 | | 0.590 | | -0.001 | | 1.182 | | 0.051 | | 0.442 | | -0.193 | | 1.077 | | 0.301 | | 0.559 | | -0.566 | | 1.684 | | 0.676 | |  |  |  |  |  |
|  | Night1-Afternoon1 | | 0.440 | | -0.152 | | 1.032 | | 0.234 | | -0.147 | | -0.782 | | 0.488 | | 0.991 | | -0.013 | | -1.138 | | 1.113 | | 1.000 | |  |  |  |  |  |
|  | Afternoon2-Afternoon1 | | 0.337 | | -0.255 | | 0.929 | | 0.527 | | -0.032 | | -0.667 | | 0.603 | | 1.000 | | -0.120 | | -1.245 | | 1.006 | | 1.000 | |  |  |  |  |  |
|  | Night2-Night1 | | -0.173 | | -0.765 | | 0.419 | | 0.966 | | -0.232 | | -0.867 | | 0.403 | | 0.900 | | 0.139 | | -0.986 | | 1.264 | | 1.000 | |  |  |  |  |  |
|  | Midday2-Predawn2 | | 0.965 | | 0.373 | | 1.557 | | **0.001** | | 0.407 | | -0.228 | | 1.042 | | 0.392 | | 0.710 | | -0.416 | | 1.835 | | 0.409 | |  |  |  |  |  |
|  | Afternoon2-Predawn2 | | 0.128 | | -0.463 | | 0.720 | | 0.994 | | 0.553 | | -0.082 | | 1.188 | | 0.112 | | -0.220 | | -1.345 | | 0.905 | | 0.997 | |  |  |  |  |  |
|  | Night2-Predawn2 | | 0.058 | | -0.533 | | 0.650 | | 1.000 | | 0.207 | | -0.428 | | 0.842 | | 0.941 | | 0.026 | | -1.099 | | 1.151 | | 1.000 | |  |  |  |  |  |
|  | Afternoon2-Midday2 | | -0.837 | | -1.428 | | -0.245 | | **0.003** | | 0.147 | | -0.488 | | 0.782 | | 0.991 | | -0.930 | | -2.055 | | 0.196 | | 0.147 | |  |  |  |  |  |
|  | Night2-Midday2 | | -0.907 | | -1.498 | | -0.315 | | **0.001** | | -0.200 | | -0.835 | | 0.435 | | 0.950 | | -0.684 | | -1.809 | | 0.442 | | 0.452 | |  |  |  |  |  |
|  | Night2-Afternoon2 | | -0.070 | | -0.662 | | 0.522 | | 1.000 | | -0.347 | | -0.982 | | 0.288 | | 0.575 | | 0.246 | | -0.879 | | 1.371 | | 0.993 | |  |  |  |  |  |
| AC | Midday1-Predawn1 | | 0.143 | | -0.942 | | 1.229 | | 1.000 | | -0.207 | | -1.030 | | 0.616 | | 0.985 | | -0.177 | | -0.713 | | 0.360 | | 0.938 | |  |  |  |  |  |
|  | Afternoon1-Predawn1 | | -0.047 | | -1.132 | | 1.039 | | 1.000 | | 0.070 | | -0.753 | | 0.893 | | 1.000 | | -0.503 | | -1.039 | | 0.034 | | 0.075 | |  |  |  |  |  |
|  | Night1-Predawn1 | | -0.223 | | -1.309 | | 0.862 | | 0.995 | | -0.023 | | -0.846 | | 0.800 | | 1.000 | | -0.427 | | -0.963 | | 0.110 | | 0.176 | |  |  |  |  |  |
|  | Predawn2-Predawn1 | | 0.012 | | -1.074 | | 1.097 | | 1.000 | | -0.148 | | -0.971 | | 0.675 | | 0.998 | | -0.297 | | -0.833 | | 0.240 | | 0.561 | |  |  |  |  |  |
|  | Afternoon1-Midday1 | | -0.190 | | -1.275 | | 0.895 | | 0.998 | | 0.277 | | -0.546 | | 1.100 | | 0.931 | | -0.326 | | -0.863 | | 0.211 | | 0.453 | |  |  |  |  |  |
|  | Night1-Midday1 | | -0.367 | | -1.452 | | 0.719 | | 0.929 | | 0.183 | | -0.640 | | 1.006 | | 0.992 | | -0.250 | | -0.787 | | 0.287 | | 0.737 | |  |  |  |  |  |
|  | Midday2-Midday1 | | 0.313 | | -0.773 | | 1.398 | | 0.968 | | -0.132 | | -0.955 | | 0.691 | | 0.999 | | 0.111 | | -0.426 | | 0.648 | | 0.995 | |  |  |  |  |  |
|  | Night1-Afternoon1 | | -0.177 | | -1.262 | | 0.909 | | 0.999 | | -0.093 | | -0.916 | | 0.730 | | 1.000 | | 0.076 | | -0.461 | | 0.613 | | 1.000 | |  |  |  |  |  |
|  | Afternoon2-Afternoon1 | | -0.100 | | -1.185 | | 0.986 | | 1.000 | | -0.005 | | -0.828 | | 0.818 | | 1.000 | | 0.037 | | -0.499 | | 0.574 | | 1.000 | |  |  |  |  |  |
|  | Night2-Night1 | | -0.225 | | -1.310 | | 0.861 | | 0.995 | | 0.285 | | -0.538 | | 1.108 | | 0.921 | | 0.148 | | -0.388 | | 0.685 | | 0.975 | |  |  |  |  |  |
|  | Midday2-Predawn2 | | 0.444 | | -0.641 | | 1.530 | | 0.837 | | -0.190 | | -1.013 | | 0.633 | | 0.991 | | 0.231 | | -0.306 | | 0.768 | | 0.802 | |  |  |  |  |  |
|  | Afternoon2-Predawn2 | | -0.158 | | -1.243 | | 0.927 | | 0.999 | | 0.213 | | -0.610 | | 1.036 | | 0.982 | | -0.168 | | -0.705 | | 0.368 | | 0.951 | |  |  |  |  |  |
|  | Night2-Predawn2 | | -0.460 | | -1.545 | | 0.626 | | 0.814 | | 0.410 | | -0.413 | | 1.233 | | 0.673 | | 0.018 | | -0.518 | | 0.555 | | 1.000 | |  |  |  |  |  |
|  | Afternoon2-Midday2 | | -0.602 | | -1.688 | | 0.483 | | 0.557 | | 0.403 | | -0.420 | | 1.226 | | 0.690 | | -0.399 | | -0.936 | | 0.137 | | 0.233 | |  |  |  |  |  |
|  | Night2-Midday2 | | -0.904 | | -1.989 | | 0.181 | | 0.141 | | 0.600 | | -0.223 | | 1.423 | | 0.253 | | -0.213 | | -0.749 | | 0.324 | | 0.857 | |  |  |  |  |  |
|  | Night2-Afternoon2 | | -0.302 | | -1.387 | | 0.784 | | 0.974 | | 0.197 | | -0.626 | | 1.020 | | 0.989 | | 0.187 | | -0.350 | | 0.724 | | 0.919 | |  |  |  |  |  |
| AM *versus* AC | ACPredawn1-AMPredawn1 | | -0.055 | | -0.991 | | 0.881 | | 1.000 | | 0.023 | | -0.764 | | 0.811 | | 1.000 | | -0.261 | | -1.206 | | 0.683 | | 0.999 | |  |  |  |  |  |
|  | ACMidday1-AMMidday1 | | 0.469 | | -0.467 | | 1.405 | | 0.876 | | 0.030 | | -0.757 | | 0.817 | | 1.000 | | -0.011 | | -0.955 | | 0.934 | | 1.000 | |  |  |  |  |  |
|  | ACAfternoon1-AMAfternoon1 | | 0.862 | | -0.074 | | 1.798 | | 0.097 | | -0.313 | | -1.101 | | 0.474 | | 0.978 | | -0.085 | | -1.030 | | 0.859 | | 1.000 | |  |  |  |  |  |
|  | ACNight1-AMNight1 | | 0.245 | | -0.691 | | 1.181 | | 1.000 | | -0.260 | | -1.047 | | 0.527 | | 0.996 | | 0.003 | | -0.941 | | 0.947 | | 1.000 | |  |  |  |  |  |
|  | ACPredawn2-AMPredawn2 | | -0.049 | | -0.985 | | 0.887 | | 1.000 | | 0.313 | | -0.474 | | 1.101 | | 0.978 | | 0.197 | | -0.747 | | 1.141 | | 1.000 | |  |  |  |  |  |
|  | ACMidday2-AMMidday2 | | -0.570 | | -1.506 | | 0.366 | | 0.654 | | -0.283 | | -1.071 | | 0.504 | | 0.991 | | -0.281 | | -1.226 | | 0.663 | | 0.999 | |  |  |  |  |  |
|  | ACAfternoon2-AMAfternoon2 | | -0.335 | | -1.271 | | 0.601 | | 0.991 | | -0.027 | | -0.814 | | 0.761 | | 1.000 | | 0.249 | | -0.695 | | 1.193 | | 1.000 | |  |  |  |  |  |
|  | ACNight2-AMNight2 | | -0.567 | | -1.503 | | 0.369 | | 0.661 | | 0.517 | | -0.271 | | 1.304 | | 0.539 | | 0.190 | | -0.755 | | 1.134 | | 1.000 | |  |  |  |  |  |

**Table S3.** Pearson’s coefficients correlation matrix between xylem sap parameters. The diverging colors indicate values ranging from -1 (red) to 1 (blue).


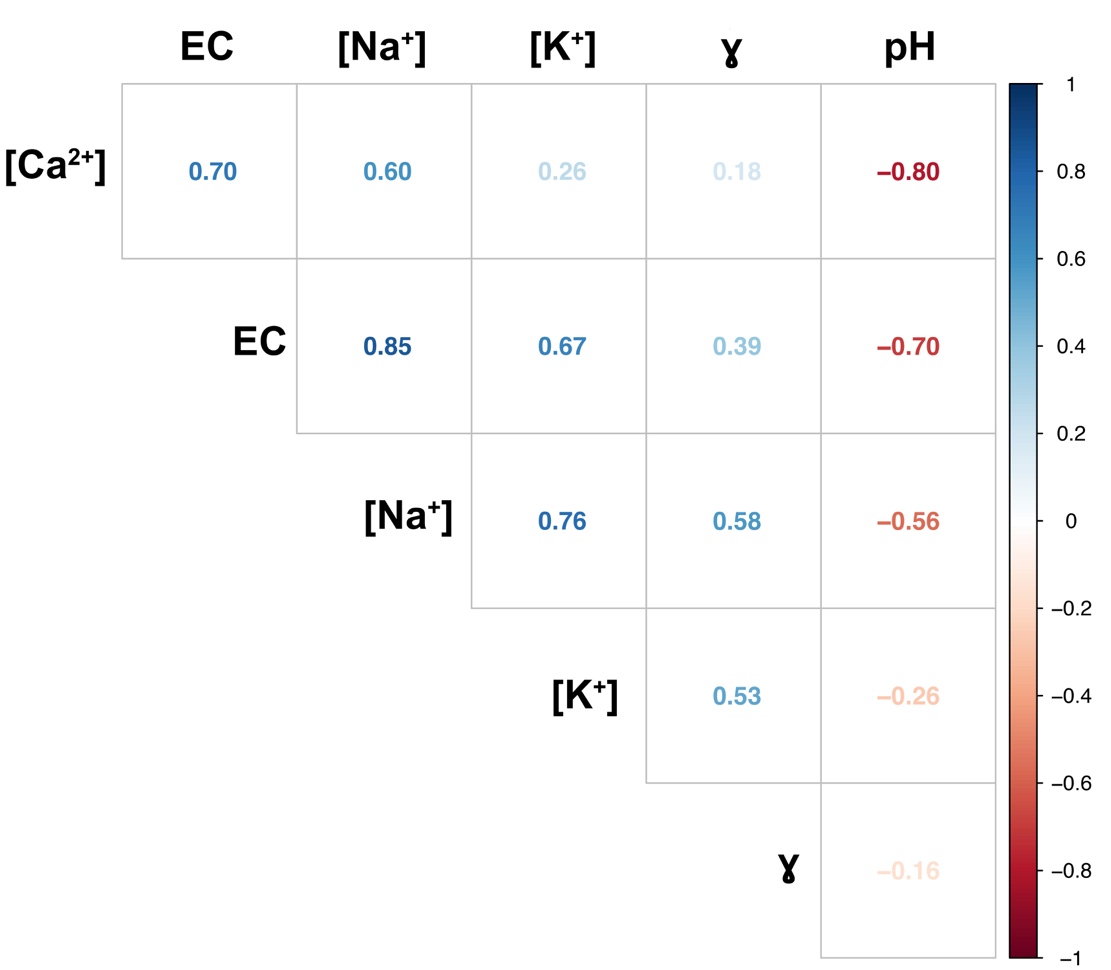


**Table S4.** Eigenvalues extracted from the principal component analysis (PCA), their variance (%), and their cumulative variance obtained by adding the successive proportions of each variance (%).

| **PCA original data** | | | |
| --- | --- | --- | --- |
|  | **Eigenvalue** | **Variance (%)** | **Cumulative variance (%)** |
| Dim.1 | 3.7455359 | 62.425599 | 62.4256 |
| Dim.2 | 1.2586351 | 20.977252 | 83.40285 |
| Dim.3 | 0.5185298 | 8.642164 | 92.04501 |
| Dim.4 | 0.206694 | 3.444899 | 95.48991 |
| Dim.5 | 0.1556735 | 2.594558 | 98.08447 |
| Dim.6 | 0.1149317 | 1.915529 | 100 |
|  |  |  |  |

**Figure S1** Tides *versus* xylem sap parameters of *Avicennia marina* ssp. *australasica*: [Na^+^] (a), [K^+^] (b), [Ca^2+^] (c), electrical conductivity (EC; d), pH (e), and surface tension (γ; f). Both quadratic (dashed lines) and linear (solid lines) functions were used to analyze relationships.


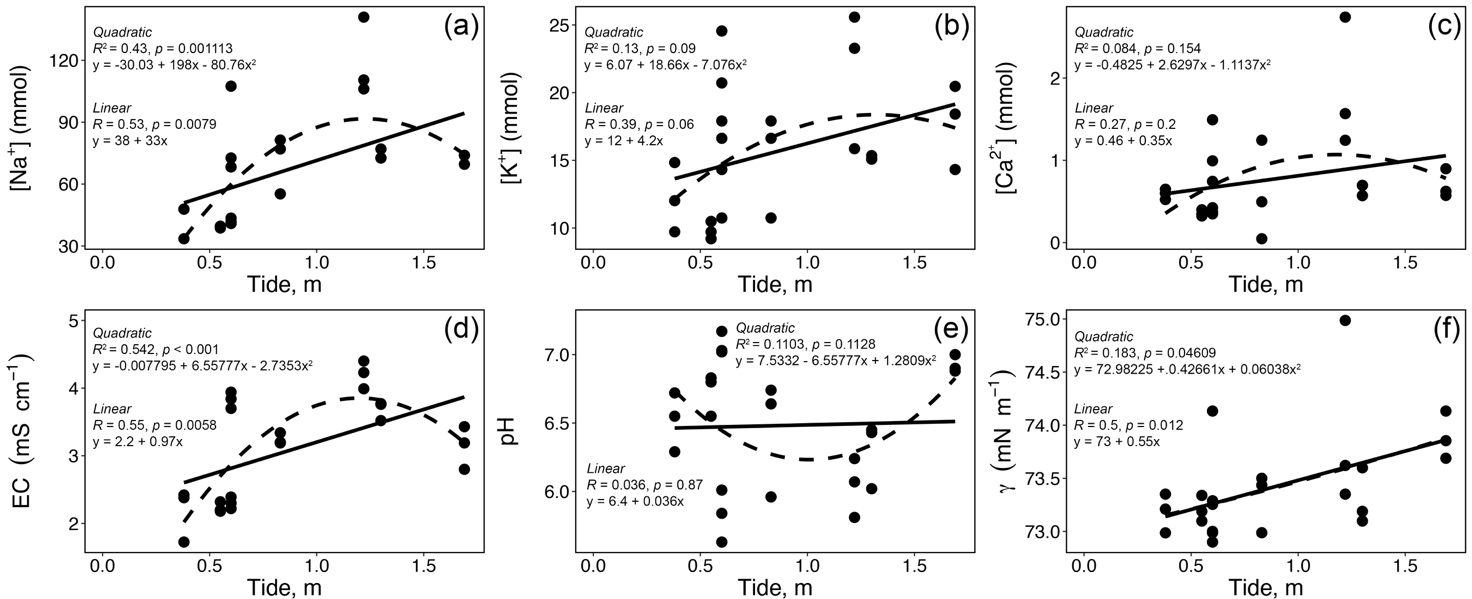


**Figure S2** Tides *versus* xylem sap parameters of *Aegiceris corniculatum*: [Na^+^] (a), [K^+^] (b), [Ca^2+^] (c), electrical conductivity (EC; d), pH (e), and surface tension (γ; f). Both quadratic (dashed lines) and linear (solid lines) functions were used to analyze relationships.


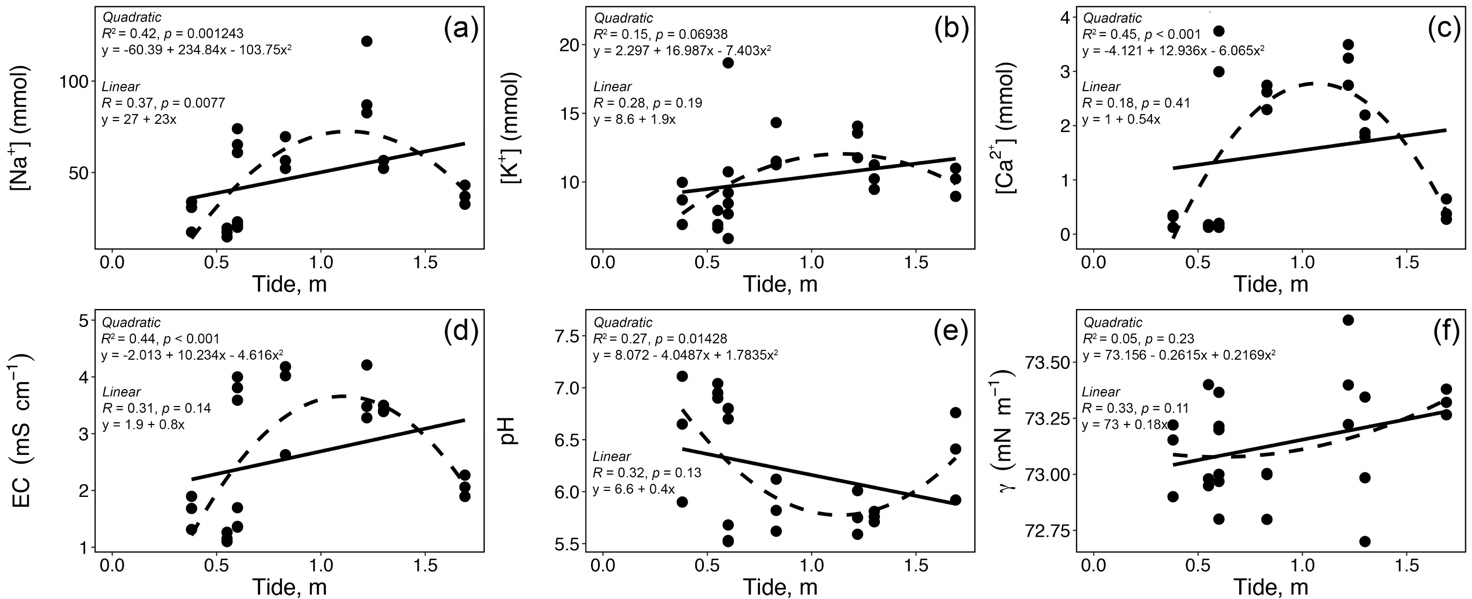

Supplement: Supplementary file 1 — Supporting information. [file PCE-48-3027-s001.docx]
